# Transverse False Tendons in the Left Ventricular Cavity Are Associated with Early Repolarization

**DOI:** 10.1371/journal.pone.0125173

**Published:** 2015-05-01

**Authors:** Yuan Liu, Ning Mi, Yiming Zhou, Peng An, Yongyi Bai, Yifang Guo, Changming Hong, Zhixin Ji, Ping Ye, Caie Wu

**Affiliations:** 1 Department of Geriatric Cardiology, Chinese PLA General Hospital, Beijing, China; 2 Clinical medicine department of Bethune medical profession sergeant school, Shijiazhuang, China; 3 Department of liver disease, Beijing General Hospital of Beijing Military Command, Beijing, China; 4 Department of internal medicine, the First People’s Hospital of Qujing, Qujing, China; 5 Department of Geriatric Cardiology, Hebei Provincial People’s Hospital, Shijiazhuang, Hebei Province, China; 6 Medical Administration, Chinese PLA General Hospital, Beijing, China; Temple University, UNITED STATES

## Abstract

**Background:**

Left ventricular false tendons (LVFTs) are related to precordial murmurs, ventricular arrhythmias and some repolarization abnormalities. Early repolarization (ER) is a specific type of repolarization abnormality.

**Objective:**

The aim of the present study was to investigate the relationship between LVFTs and ER.

**Methods:**

This study retrospectively included 99 consecutive healthy subjects and 33 patients with ER. Early repolarization was defined as an elevation of the QRS-ST junction of >0.1 mV from baseline in at least 2 inferior or lateral leads, manifested as QRS slurring or notching. Each participant was examined using echocardiography with second harmonic imaging, and the attachments of the LVFTs were recorded.

**Results:**

A total of 93 LVFTs were present in 82 (83%) of the 99 healthy subjects. Of these 93 LVFTs, the majority (79/93, or 84.9%) were longitudinal-type LVFTs, which originated from the basal interventricular septum (IVS) and progressed toward the apical segment of the left ventricular free wall. There were significant differences in the positioning of the LVFTs between the ER patients and control (P < 0.0001). LVFTs between mid-IVS to the middle of the LV free wall were found more common in patients with ER compared with control subjects (47.5% vs. 6.5%, P < 0.0001). In the ER group, LVFTs between the basal IVS to the apical segment of LV free wall were only identified in 21% of the LVFTs, compared to a value of 84.9% for the control group (P < 0.0001). The distribution of LVFT trends in the ER group was also significantly different from that in the control group (P < 0.05).

**Conclusions:**

LVFTs are commonly visualized using echocardiography. An LVFT from the basal IVS to the apical segment of the left ventricular free wall may be a normal anatomical structure in the left ventricular cavity. On the contrary, transverse false tendons in the left ventricular cavity may be associated with ER.

## Introduction

Left ventricular false tendons (LVFTs), also known as left ventricular fibromuscular bands or malposition tendons, are discrete fibromuscular structures of varying length and thickness [1–5]. These structures span the left ventricular cavity to connect distant sites; on an endocardium, these structures appear beside the chordae tendineae. In general, LVFTs contain conductive tissue; an exception is the less common, thinner, oyster-white LVFT, which consists entirely of dense connective tissue. A few myocardial cells are diffusely distributed within LVFTs [6–8]. This inner conductive tissue might be an extending part of the His bundle that distributes through the ventricular wall to coordinate left ventricular contraction and relaxation [9].

LVFTs are commonly visualized using echocardiography and at autopsy [5,9–12]. In 1896, Turner first described LVFTs by autopsy [13], but the role of LVFTs was not investigated due to the lack of noninvasive equipment. The application of ultrasound diagnostic instruments in medicine has made it possible to detect LVFTs noninvasively. Nishimura published the first paper using echocardiography to study LVFTs in 1981 [14], and the fundamental and clinical research regarding LVFTs peaked by the end of the 1980s. Following the recent advent of electrophysiological and interventional technologies, researchers have again turned their attention to LVFTs. Although LVFTs can be readily identified with routine two-dimensional echocardiography, the echocardiographic detection rates vary widely from 0.5–70% due to several factors, including operator skill, the equipment used, and knowledge of these phenomena. Thus, there is a need for a large-scale investigation of the prevalence of LVFTs.

Although LVFTs are considered by many to be benign anatomical variants [4,15], several diseases have been reported to be associated with LVFTs. This includes an increased prevalence of innocent precordial murmur [4,16,17], ventricular arrhythmia [16,17], and giant inverse T waves and left ventricular hypertrophy evident on resting electrocardiograms [18–20].

The J-point on the electrocardiographic waveform is historically defined as the junction between the end of the QRS complex and the beginning of the ST-segment [21–22]. The normal variants of the complex of J-points and J-waves in young subjects were defined by the term ‘early repolarization’ (ER). The J-point elevations in ER have been previously considered a universally benign normal variant. In more recent years, the variations in the electrocardiographic patterns of J-point elevations, and the complex of J-points and J-waves in ER, in conjunction with disparities in associated sudden cardiac death (SCD) risk, have lead to recognition of the need to carefully classify the spectrum of these observations [23]. Many questions about the pathogenesis of J-wave patterns, and the associated magnitudes of risk, remain unanswered [24].

Based on previously published observations [19,25], we hypothesized that the existence of an LVFT may be an important mechanism in ER. The objectives of our study were to investigate the prevalence of LVFTs, to describe the echocardiographic characteristics of LVFTs, and to investigate the association between LVFTs and ER.

## Materials and Methods

This study was approved by the ethics committee of Chinese PLA General Hospital and Clinical medicine department of Bethune medical profession sergeant school (Approval No. PLA204015). All of the participants reported themselves to be of Han nationality and provided written informed consent.

### Subjects

We performed a case-control study investigating LVFT characteristics and the relationship between LVFTs and ER in inpatient and outpatient from November 1999 through November 2007 from Chinese PLA General Hospital and Hebei Medical University. Resting 12-lead ECGs were ordered by healthcare providers for standard clinical indications, usually to screen for occult disease and to obtain a baseline when care is initiated. The VA Palo Alto Health Care System has used a centralized, computerized ECG system for the collection, storage, and analysis of ECGs (GE Healthcare, Wauwatosa, WI). ER was defined as an elevation of the QRS-ST junction of >0.1 mV from baseline in at least 2 inferior or lateral leads, manifested as QRS slurring or notching. All ECGs exhibiting ST-segment elevation as determined by the computer measurements were reread by 3 observers (blinded to outcomes) and corrected when necessary (5.6%). For patients with multiple ECGs, only the first ECG was considered. Only subjects with ER were included in the patient group. The criteria for diagnosis of ER are those as described by Miyazaki et al. 2010 [26], namely J-point elevation manifested either as QRS slurring (at the transition from the QRS segment to the ST segment) or notching (a positive deflection inscribed on terminal S wave), ST-segment elevation with upper concavity and prominent T waves in at least 2 contiguous leads. The exclusion criteria included electrolyte disturbances, acute myocardial infarction, acute pericarditis, esophageal hiatal hernia, diaphragmatocele, hydrothorax, myocardial bridge.

Control subjects were selected from among inpatients with minor illnesses from the ophthalmology, gastroenterology, otorhinolaryngology, and orthopedics departments and from community-based inhabitants who were free of cardiovascular disease. These participants were subject to the same exclusion criteria as the case subjects.

Eventually 33 patients with ER and 99 controls without ES (as control 1) were recruited. Forty-four of age- and sex- matching participants (matched ER group, as control 2) were randomized selected from 99 subjects above by simple randomization method.

### Determination of left ventricular false tendons

All echocardiography examinations were performed using a Philips SONOS 5500 equipped with a 2.5-MHz linear-array and a second harmonic transducer. Several standard views were employed: parasternal long and short axis views, apical and subcostal four-chamber views, and apical long-axis and two-chamber views. The diagnosis of an LVFT was based on the finding of a distinctive linear echogenic strand not related to the mitral valve apparatus. The echogenic strand had to be traversing the LV cavity and connecting the LV free wall or papillary muscle with the ventricular septum, identified in at least two echocardiographic planes of view. All equivocal interpretations (such as when an LVFT was noted in only one echocardiographic view) were reviewed by a second experienced echocardiographer, and an LVFT was considered present only if both echocardiographers agreed. Particular care was taken to differentiate LVFTs from other entities, such as thickened ventricular trabeculations, intraventricular masses and/or tumors, a discrete subaortic membrane, an accessory anterior mitral leaflet, or a flail mitral valve with ruptured chordae tendineae. To improve our visualization of the LVFTs, the probe was often turned slightly upward and downward, as multi-plane and multi-degree images are known to enhance the visualization of LVFTs.

### Echocardiographic measurements in the ER and control groups

The diameters of the left atrium (LA), left ventricle (LV) and right ventricle (RV), and the thicknesses of the anterior wall of the right ventricle (RVAW) and the posterior wall of left ventricle (LVPW) were measured. The long-axis diameter of the left ventricle at end-systole (LVDs1) and end-diastole (LVDd1) was also measured, from an apical four-chamber view. In addition, the anteroposterior diameter of the left ventricle at end-systole (LVDs2) and end-diastole (LVDd2) was measured, from a parasternal long-axis view. From these measurements, the ratios of (LVDd2-LVDs2)/LVDd2 and (LVDd1-LVDs1)/LVDd1 were calculated.

### Echocardiographic characterization of left ventricular false tendons

Based on the attachments of the LVFTs in the apical-, mid-, or basal-third segments along the long axis of the left ventricle, we classified each LVFT into various types. The LVFTs were classified as either longitudinal (≤ 45^°^) or transverse (> 45^°^) by visual measurement.

### Statistical analyses

The chi-squared (χ^2^) test was used to compare the rates of left ventricular false tendons between the two groups. We compared the echocardiographic characteristics of the LVFTs between the participants with and without ER using general linear models (least-squares means) for continuous variables and logistic regression models for categorical variables. Statistical analyses were performed using Stata software version 11.0 (Stata Corporation, College Station, TX, USA). A two-sided value of P < 0.05 was considered significant.

## Results

### General characteristics of the subject and control groups

The participants consisted of 99 healthy subjects (control 1 group), 44 of age- and gender-match patients (matched ER group, control 2), and 33 patients with ER. The general characteristics of the subjects, control 1 and control 2 groups are presented in Table 1. Compared with the control 1 or control 2 group, the ER group showed a significantly greater incidence of premature ventricular contractions, a significantly slower heart rate, and a longer QTd in the ST elevation leads (detected through resting 12-lead ECGs, Table 1). In addition, the incidence of chest pain and dyspnea were higher in the ER group (Table 1). There were no significant differences in the LA, LV and RV diameters, RVAW and LVPW thicknesses, or the (LVDd-LVDs)/LVDd values between the groups. The (LVDd-LVDs)/LVDd value for the left ventricular long axis (obtained from the apical four-chamber view) was higher than that for the left ventricular short axis in both groups.

**Table 1:** General characteristics and echocardiographic parameters of the ER, control 1 and 2 groups.

	ER (n = 33)	Control 1 (n = 99)	Control 2 (n = 44)		P
Male/Female	32/1	66/33		42/2	
Dyspnea (n)	25	12		7	P < 0.001
Chest pain (n)	11	8		6	P < 0.001
Premature ventricular contraction (n)	16	4		3	P < 0.001
Heart rate (beats/min)	61.9 ± 7.4	76.8 ± 8.9		77.3 ± 8.7	P < 0.05
QTd (ms)	427 ± 28	411 ± 19		411 ± 21	P > 0.05
QTd in ST elevation leads (ms)	448 ± 21^*^				
LA diameter	34.2 ± 4.6	33.5 ± 5.1		33.8 ± 4.7	P > 0.05
LV diameter	45.1 ± 4.7	50.3 ± 5.9		49.3 ± 4.6	P > 0.05
RV diameter	16.6 ± 3.9	15.8 ± 4.3		16.9 ± 3.9	P > 0.05
RVAW thickness	4.3 ± 1.0	4.5 ± 1.2		4.5 ± 1.2	P > 0.05
LVPW thickness	8.9 ± 1.8	8.4 ± 2.2		8.3 ± 2.2	P > 0.05
RVAW/LVPW ratio	0.49 ± 0.09	0.55 ± 0.12		0.53 ± 0.13	P = 0.05
(LVDd-LVDs)/LVDd					P > 0.05
Short axis	0.37 ± 0.04	0.36 ± 0.06		0.35 ± 0.05	P > 0.05
Long axis	0.14 ± 0.03^#^	0.15 ± 0.03^#^		0.15 ± 0.03^#^	P > 0.05

ER, early repolarization syndrome;

^*^ P < 0.05, compared with QTd in the control 1 or Control 2 group.

^#^ P < 0.01, compared with the short axis value.

Control 2 group: age-and gender- matching patients from control_1_ group with ER group.

### The presence of LVFTs

Of the 99 healthy participants, 93 LVFTs were confirmed in 82 (82.8%) and were absent in 17 (17.2%). Among the patients with ER, LVFTs were confirmed in 30 (90.9%) of the 33 cases. There were no significant differences between males and females in the detection rate of LVFTs, and no significant differences were found regarding the presence of LVFTs between the ER group and the control groups (See Tables 1, 2 and 3).

**Table 2:** LVFT characteristics in the control 1 and ER groups.

Group	Control l group			ER
	Total	Male	Female	Male/female
Number of cases	99	66	33	32/1
Age (years)	31.6 ± 7.2			33.6 ± 9.8
*LVFT*				
Number of cases	82	42	40	30
Number of LVFTs	93	50	43	38
*Position*, n(%)				^*^
Type A	6 (6.5%)	3	3	18 (47.5%)
Type B	79 (84.9%)	42	37	8 (21%)
Type C	6 (6.5%)	4	2	4 (10.5%)
Other	2 (2.1%)	1	1	8 (21%)
*Trends*				*
Longitudinal	86 (92.6%)	45	41	20
Transverse	7 (8.4%)	5	2	18

Type A, connection between the mid-IVS to the middle of the left ventricular free wall; Type B, attachment between the basal IVS to the apical segment of the left ventricular free wall; Type C, connection between the mid-IVS to the apical segment of the left ventricular free wall; Other, not classified as Types A, B or C. ^*^P < 0.05, compared to the control.

**Table 3:** LVFT characteristics in the control 2 and ER groups.

	Total	Control 2 group		ER group
		Male	Female	Male /female
Number of cases	44	42	2	32/1
Age (years)	32.3 ± 5.9	32.3 ± 5.9	32.0 ± 0.0	33.6 ± 9.8
*LVFT*				
Number of cases	39	37	2	30
Number of LVFTs	43	41	2	38
*Position*, n(%)				
Type A	2 (4.7%)	2(4.9%)	0	18 (47.5%)
Type B	36 (83.7%)	34(82.9%)	2(100%)	8 (21%)
Type C	4 (9.3%)	4(9.8%)	0	4 (10.5%)
Other	1 (2.3%)	1(2.4%)	0	8 (21%)
*Trends*, n(%)				
Longitudinal	40 (92.6%)	38(92.7%)	2(100%)	20(52.6%)
Transverse	3 (8.4%)	3(7.3%)	0	18(47.4%)

### Echocardiographic characterization of LVFTs

LVFTs were typically visualized in the apical four-chamber view, the apical two-chamber view, and either the non-standard parasternal left ventricular long-axis or the apical long-axis view.

According to their attachments, we classified the LVFTs into four types: Type A was defined as an attachment between the mid-interventricular septum (IVS) to the middle of the left ventricular free wall; Type B was defined as an attachment between the basal IVS to the apical segment of the left ventricular free wall; Type C was defined as an attachment between the mid-IVS to the apical segment of the left ventricular free wall; and “Other” was defined to include LVFTs not classified as Types A, B or C. Of the 93 LVFTs in the 82 healthy participants, the most commonly visualized position was from the basal IVS to the apical segment of the left ventricular free wall (Type B), comprising 84.9% (79/93) of all LVFTs in the 82 healthy subjects, and found in 79 of these 82 cases (96%). The other positions included the area from the mid-IVS to the middle of the left ventricular free wall (Type A) and the area from the mid-IVS to the apical segment of the left ventricular free wall (Type C).

### LVFTs in patients with ER

Compared with the control 1 group, a significant difference in the ratio of males and females was observed in the group of patients with ER (male/female, ER vs. control_1_ group: 32/1 vs. 66/33, P < 0.0005). The presence of LVFTs in the ER group was not significantly different when compared with controls (P > 0.05); however, there were significant differences in the positioning of the LVFTs between the two groups (P < 0.0001), with Type A LVFTs more common in patients with ER compared with control 1 subjects (47.5% vs. 6.5%). In the ER group, Type B LVFTs were only identified in 21% of the LVFTs, compared to a value of 84.9% for the control group. The distribution of LVFT trends in the ER group was also significantly different from that in the control 1 group (P < 0.05) (Table 2). In the age- and gender- match control 2 group, these result were similar to control1 group compared with ER group (Table 3)

## Discussion

### Characteristics of the ER patient group

The ratio of males to females in the ER group was 32:1, which suggests that there may be a repolarization difference between males and females with ER. This observation is consistent with previous findings that testosterone may influence the mechanisms underlying repolarization [27]. In addition, the QT interval in the leads showing ST segment elevation was longer in the ER subjects compared to controls, which demonstrates unevenness in the repolarization in ER patients. A dispersion of repolarization is known to underlie the genesis of certain arrhythmias, and may explain the higher incidence of premature ventricular contraction (detected through resting 12-lead ECG) in the ER group compared with the control group [28].

The increased incidence of dyspnea and chest pain in the ER group, compared with the control group, is an interesting observation. However, this may simply be a reflection of the enrollment procedure, namely that the patients in the ER group would be expected to have a higher incidence of dyspnea and chest pain than those enrolled in the control group, who were either inpatients with minor illnesses from ophthalmology, gastroenterology, otorhinolaryngology or orthopedics wards, or community-based inhabitants free of cardiovascular disease.

### The prevalence of LVFTs in the control group

In this study, we found that the detection rate of LVFTs was higher in the control group than was previously reported [3]. This discrepancy may be explained by considering the following three aspects. First, there has been a long debate regarding the pathological nature of LVFTs, resulting in most LVFTs being ignored by the echocardiographer. Second, the detection rate of LVFTs is low because LVFTs require additional planes and/or angles to be detected. Third, the development of echocardiography technologies such as second harmonic wave imaging and incremental frame frequency has increased the detection rate of LVFTs.

### The constituent ratio of LVFT position in the healthy subject group

The number of LVFTs connecting the basal IVS to the apical segment of the left ventricular free wall was the most common position in the healthy subjects (84.9% of all LVFTs). Of the 82 cases with LVFTs, 79 had this type of LVFT (96.3%). The readily detectable positions were upward or downward, the probe was rotated slightly along the parasternal left ventricular long-axis view. Thus, we hypothesize that the LVFT from the basal IVS to the apical segment of the left ventricular free wall is a normal anatomical structure that participates in electrocardiographic conduction and helps to maintain the functional status of the left ventricular free wall.

### The relationship between LVFT and ER

The relationship between LVFT and abnormal repolarization has been investigated in several studies. Salazar [19] reported a child with considerable electrocardiographic repolarization who showed an elevated ST segment with a negative T wave in the anterolateral and inferior leads. The patient was diagnosed with an anomalous LVFT that was 6 mm in diameter. In our study, we found that the prevalence of LVFTs among ER patients was not significantly different to that of healthy participants. This finding suggests that LVFTs may not be specific to ER; however, when we further analyzed the features of the LVFTs in the ER group, we found that the incidence of transverse LVFTs was higher than that of longitudinal LVFTs, and that the majority of LVFTs connected the mid-IVS to the middle of the left ventricular free wall. Conversely, we found that the minority of LVFTs connected the basal IVS to the apical segment of the left ventricular free wall. Furthermore, the angle formed by the LVFT and the IVS was larger in the ER group than in the control group, suggesting a relationship between transversal LVFTs and ER. Thus, the position and trend of the LVFT may be correlated with repolarization. There are several possible mechanisms that might explain this phenomenon. We propose that for an LVFT with an insertion in the middle of the left ventricular free wall with a larger angle, the attachment of the LVFT may be stretched such that the regional myocardium becomes stretched beyond its normal limit, because the transverse relaxation of the left ventricular free wall is greater than that of the longitudinal direction. Stretched myocardial cells repolarize earlier than normal, thereby resulting in a myocardial voltage gradient between the endocardium and epicardium and an elevated ST segment [29]. In contrast, in cases in which the insertion was in an apical segment of the left ventricular free wall at a smaller angle, the attachments of longitudinal LVFTs may not have been substantially stretched. In these cases, the regional myocardium would only be stretched to a small extent because relaxation of the left ventricular free wall in the longitudinal direction was small compared to that in the transverse direction.

The present study has limitations. There is no mechanistic analysis regarding the association between transverse LVFT and ER because the present study did not perform any electrophysiological investigations except electrocardiography. Additional electrophysiological studies (EPS) may be helpful to confirm the relationship between LVETs and ER. However, in the present study, EPS, which is an invasive procedure, were not approved to perform in healthy or apparently healthy participants (most of the ER subjects did not have a history of aborted sudden death), which is an unavoidable limitation of the present study. Alternatively, we have designed a study to investigate the relationship between LVFT, ER and inducible ventricular fibrillation (VF) in patients underwent EPS, which may complement results of the present study.

In summary, LVFTs are not specific to ER, but the position and trend of the LVFT has an effect on repolarization. This finding is especially true for transverse LVFTs. Closely monitoring the characteristics of LVFTs may help elucidate the mechanism of repolarization in ER and lead to viable treatment options. In addition, echocardiographic detection of LVFTs is associated with the presence of ER.
